# Zinc Biofortification in *Vitis vinifera*: Implications for Quality and Wine Production

**DOI:** 10.3390/plants11182442

**Published:** 2022-09-19

**Authors:** Diana Daccak, Fernando C. Lidon, Inês Carmo Luís, Ana Coelho Marques, Ana Rita F. Coelho, Cláudia Campos Pessoa, João Caleiro, José C. Ramalho, António E. Leitão, Maria José Silva, Ana Paula Rodrigues, Mauro Guerra, Roberta G. Leitão, Paula Scotti Campos, Isabel P. Pais, José N. Semedo, Nuno Alvarenga, Elsa M. Gonçalves, Maria Manuela Silva, Paulo Legoinha, Carlos Galhano, José Carlos Kullberg, Maria Brito, Manuela Simões, Maria Fernanda Pessoa, Fernando H. Reboredo

**Affiliations:** 1 Earth Sciences Department, Faculdade de Ciências e Tecnologia, Campus da Caparica, Universidade Nova de Lisboa, 2829-516 Caparica, Portugal; 2 GeoBiotec Research Center, Faculdade de Ciências e Tecnologia, Campus da Caparica, Universidade Nova de Lisboa, 2829-516 Caparica, Portugal; 3 PlantStress & Biodiversity Laboratory, Centro de Estudos Florestais (CEF), Instituto Superior Agronomia (ISA), Universidade de Lisboa (ULisboa), Quinta do Marquês, Av. República, 2784-505, Oeiras and Tapada da Ajuda, 1349-017 Lisboa, Portugal; 4 LIBPhys, Physics Department, Faculdade de Ciências e Tecnologia, Universidade Nova de Lisboa, Campus da Caparica, 2829-516 Caparica, Portugal; 5 Instituto Nacional de Investigação Agrária e Veterinária, I.P. (INIAV), Avenida da República, Quinta do Marquês, 2780-157 Oeiras, Portugal; 6 Escola Superior de Educação Almeida Garrett (ESEAG-COFAC), Avenida do Campo Grande 376, 1749-024 Lisboa, Portugal

**Keywords:** grape’s quality, variety fernão pires, winemaking, zn agronomic biofortification

## Abstract

Nowadays, there is a growing concern about micronutrient deficits in food products, with agronomic biofortification being considered a mitigation strategy. In this context, as Zn is essential for growth and maintenance of human health, a workflow for the biofortification of grapes from the *Vitis vinifera* variety Fernão Pires, which contains this nutrient, was carried out considering the soil properties of the vineyard. Additionally, Zn accumulation in the tissues of the grapes and the implications for some quality parameters and on winemaking were assessed. Vines were sprayed three times with ZnO and ZnSO_4_ at concentrations of 150, 450, and 900 g ha^−1^ during the production cycle. Physiological data were obtained through chlorophyll a fluorescence data, to access the potential symptoms of toxicity. At harvest, treated grapes revealed significant increases of Zn concentration relative to the control, being more pronounced for ZnO and ZnSO_4_ in the skin and seeds, respectively. After winemaking, an increase was also found regarding the control (i.e., 1.59-fold with ZnSO_4_-450 g ha^−1^). The contents of the sugars and fatty acids, as well as the colorimetric analyses, were also assessed, but significant variations were not found among treatments. In general, Zn biofortification increased with ZnO and ZnSO_4_, without significantly affecting the physicochemical characteristics of grapes.

## 1. Introduction

Zinc is the second-most-common transition metal in living organisms [1], being essential for the metabolism of humans and crops. Zinc has several physiological functions, namely in enzyme kinetics, cell membrane integrity, control of oxy radicals, and synthesis of sugars and chlorophylls [2,3]. Regarding enzymes, Zn is a cofactor of carbonic anhydrase, carboxypeptidase, RNA polymerase, and Zn-superoxide dismutase [2,4]; these enzymes are involved in the proteosynthesis and metabolism of carbohydrates, lipids, and nucleic acids [4]. Zinc ions are also involved in the transcription factor family—called zinc fingers, controlling the proliferation and differentiation of cells [4].

Optimal growth of most crops requires Zn concentrations ranging between 15 and 20 mg kg^−1^_DW_ [1,2]. Nevertheless, when the Zn threshold is surpassed, symptoms of toxicity can occur, such as decrease in roots and shoots growth, metabolism deviation, and oxidative damage [5,6]. Chlorosis develops in the younger leaves extending to the other leaves if the toxicity persists, and an inhibition of photosystems I and II can occur reversibly if not subjected to a constant stress [6]. Interference with the ionic balance can also occur [7]. Among different plant species, an approximate value for the threshold of Zn toxicity in leaves is about 300 μg/g_DW_ [1].

Regarding human health, Zn is found in several tissues, such as the muscles, bones, liver, and brain (i.e., synaptic vesicles) [8]. Moreover, Zn deficiency has a foreshadowed effect in the epidermal, nervous, gastrointestinal, skeletal, immune, and reproductive systems. Likewise, its deficiency triggers many human health problems, for instance an inhibition in growth, a weakened immunity system, and an increase in the risk of infections, disorders of the gastrointestinal or urinary tract, and cancer [8,9,10].

According to estimates, 17.3% of the world’s population is at risk of inadequate Zn intake. Country-specific estimated prevalence of inadequate Zn intake was correlated negatively with the total energy, the Zn contents of the national food supply, and the percentual value of Zn derived from foods of animal origin and was correlated positively with the phytate:zinc molar ratio of the food supply [11]. This inadequacy may be fairly common, particularly in Sub-Saharan Africa and South Asia, allowing inter-country comparisons and existing primarily where Zn deficiency is more acute.

In this context, biofortification programs of different crops mostly used in human consumption have been implemented worldwide. Regarding rice, four different Zn forms were applied as a foliar treatment on three cultivars under a field trial, and Zn bioavailability was assessed by in vitro digestion/Caco-2 cell model [12]. It was observed that foliar Zn fertilization promote grain Zn concentration and Zn bioavailability among the rice cultivars, especially in the case of Zn-amino acid and ZnSO_4_. On average, Zn-amino acid and ZnSO_4_ increased Zn concentration in polished rice up to 24.04% and 22.47%, respectively. Furthermore, foliar Zn application could maintain grain yield and the protein and minerals (Fe and Ca) quality of the polished rice. [12]. Moreover, through the construction of plant transformation vectors and transgenic indica rice plants, it was possible to reach approximately 30% of the estimated average requirement (EAR) of Fe and Zn in the human diet, without a yield reduction, when it is common to detect concentrations of approximately 2 μg g^−1^ Fe and 16 μg g^−1 ^ Zn in polished grains of different rice varieties. The HarvestPlus breeding programs for biofortified rice target 13 μg g^−1  ^Fe and 28 μg g^−1  ^Zn levels, c.a. 30% of the EAR [13].

The biofortification of *Pisum sativum* through Zn application in soils at concentrations of 4 and 8 mg ZnSO_4_·7H_2_O kg^−1^ and foliar Zn application of two sprays of 0.25% or 0.5% (*w/v*) ZnSO_4_·7H_2_O before flowering and at early grain-filling stage was studied. Foliar application prompted increases above 60 mg Zn kg^−1^ in grain, while during the cooking process a decrease of *c.a.* 30% in grain Zn concentration was noted [14]. The *Triticum aestivum* L. biofortification program at the International Maize and Wheat Improvement Center (CIMMYT) led to a partnership to breed competitive wheat varieties with 40% higher Zn concentration (>12 mg/kg) over the commercial varieties in the target regions of South Asia [15,16]. Biofortification of high-yielding maize varieties in Zn is of great importance to the health of those whose diets are mostly based on this staple crop, with the USA and China being the world’s biggest maize consumers. Clinical studies have so far indicated that genetically biofortified maize increased Zn absorption in human bodies [17], which is quite relevant regarding the adequate daily Zn intake.

It is well-established that foliar spraying is the best approach to increase the level of Zn, as well as the levels of other nutrients, in edible plant tissues, when compared with soil fertilization [12,14,18]. Plus, foliar spraying does not depend upon root-to-shoot translocation [19,20]. Nevertheless, some authors use a double fertilization through the foliar application of organic fertilizer, without significantly affecting the vine vigor and fruit quality of grapes, while impacting the ionome and phenolic compounds [21]. Accordingly, Zn biofortification of grapes of *Vitis vinifera* L. variety Fernão Pires through foliar spraying with ZnSO_4_ and ZnO prompt this study, further motivation being to assess the physicochemical attributes of the fruits and winemaking.

## 2. Results

The contents of mineral elements in the soil of the vineyard showed the following pattern: K > Ca > Fe > P > S > Mn > Zn (Table 1). The organic matter revealed a mean value of 1.36% (Table 1). The electrical conductivity, which is closely linked to the salt concentration that affects the energy expenditure used for water absorption (osmotic effect) by the roots, presented a value of 93.4 μS cm ^−1^ (Table 1). The pH was slightly acidic, being suitable for vines production (i.e., 6.85) (Table 1).

### 2.1. Physiological Monitoring of Photossintetic Parameters during Biofortification with Zn

**Figure 1:**
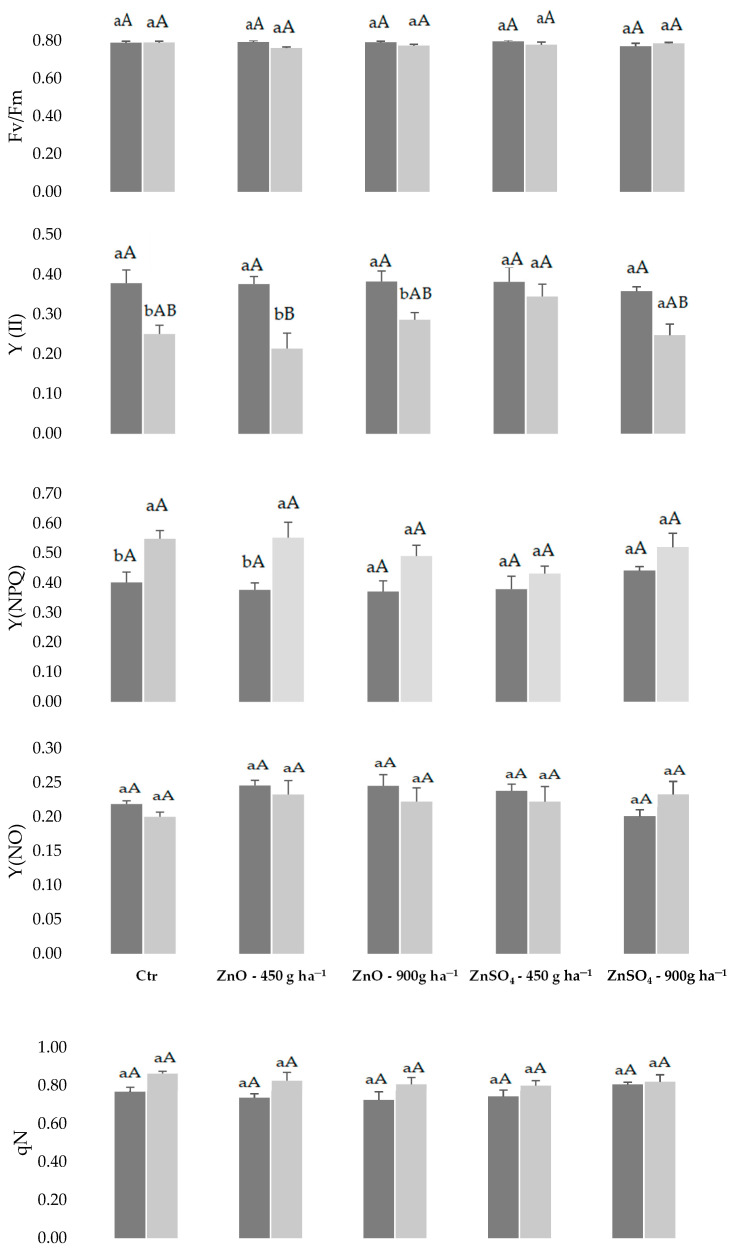

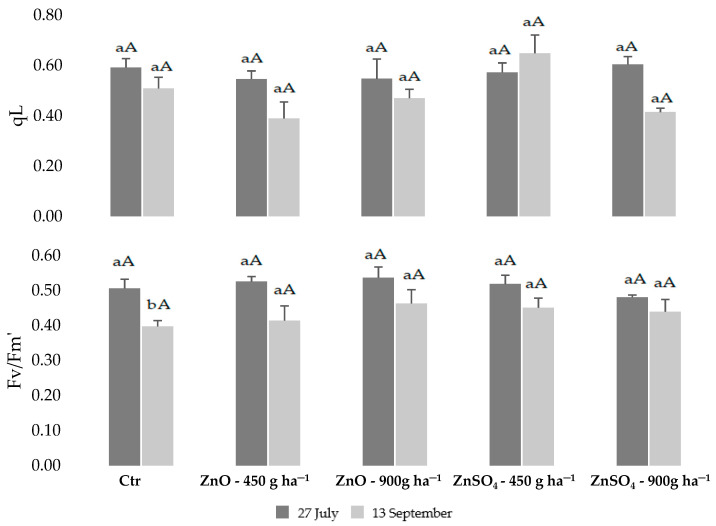
Mean values ± SE of chlorophyll a fluorescence, maximal photochemical efficiency of PSII (Fv/Fm), estimate of the quantum yield of photosynthetic noncyclic electron transport (Y(_II_)), estimate of the quantum yield of regulated energy dissipation (Y(_NPQ_)), and nonregulated energy (heat and fluorescence) dissipation (Y(_NO_)) of PSII, coefficient of non-photochemical (qN) and photochemical (qL) fluorescence quenching, and actual PSII efficiency of energy conversion under light (Fv’/Fm’) in leaves of *Vitis vinifera* L. variety Fernão Pires, submitted to Zn biofortification, on 27 July 2018 and 13 September 2018 (after the 3rd foliar spray). For each parameter, the mean values ± SE (n = 6) followed by different letters express significant differences among analytical dates for each treatment (a, b), or among treatments for the same analytical date (A, B, (statistical analysis using the single-factor ANOVA test, *p* ≤ 0.05).

Regarding the physiological response of *Vitis vinifera* L. variety Fernão Pires to Zn biofortification, the maximum efficiency of PSII (Fv/Fm) denotes the absence of significant impact among treatments and between two analyzed moments, revealing maintenance of a high efficiency (Figure 1).

Relatively to the set of estimates of the quantum transport yields, the unregulated dissipation (heat and fluorescence-Y(_NO_)) did not show a relevant variation between treatments and dates. Besides, for the photosynthetic non-cyclic electron transport (Y(_II_)) and regulated energy dissipation (Y(_NPQ_)), among treatments there were no significant differences relative to the control. However, between dates some significant variations were observed, namely a decrease for the control and ZnO treatments in the Y(II) and an increase in the control and ZnO 450 g ha^−1^ in Y (_NPQ_) in the second assessment (Figure 1).

Considering the proportion of energy dissipated, as heat, by photoprotective mechanisms (qN) and by energy captured by the PSII open reaction centers, which was used for photochemical events (qL), relevant differences between the treatments (ZnO and ZnSO_4_) were not found relatively to control and between both dates (Figure 1). The analysis of qN corroborates the variation of Y(_NPQ_), since both parameters increased between the evaluation dates, still with no obvious differences between treatments. Relative to the control, the proportion of energy captured by the PSII open reaction centers and used for photochemical events (qL) tended to decrease among treatments on both dates (except for the treatment of 900 g ha^−1^ of ZnSO_4_ on the first date evaluation and ZnSO_4_ treatment of 450 g ha^−1^ on the second evaluation date, as shown in Figure 1.

The photochemical efficiency of PSII (Fv′/Fm′), relative to the control, showed that plants with foliar application tended to have higher values on both dates (although not significant), which implies that the functioning of PSII was not affected by Zn biofortification (Figure 1).

### 2.2. Zinc Content in Leaves, Grapes, and Wine

Relative to the control, after the third foliar application with both Zn fertilizers (ZnO and ZnSO_4_), significantly higher values of Zn in all treatments were found in grapes (except for 150 g ha^−1^ of ZnSO_4_—Table 2). Besides, Zn concentration in the leaves augmented in the higher concentrations of both treatments (Table 2).

At harvest, the seed and skin revealed a significant increase in all the grapes submitted to Zn treatments (except for the seed in treatments 150 g ha^−1^ of ZnO and 900 g ha^−1^ of ZnSO_4_, although in this last case with a superior amount relative to the control) (Table 2). Comparing both tissues, Zn accumulation occurred mainly in the skin, presenting a higher value with 900 g ha^−1^-ZnO (ca. 2.54-fold increase), while in the seed 150 g ha^−1^-ZnSO_4_ revealed a higher content (ca. 2.03-fold increase) (Table 2).

Considering the winemaking process, compared to the control, all the Zn biofortified grapes led to a wine with a significant increase in the Zn amount (up to 1.59-fold increase—Table 2).

### 2.3. Sugar Composition after Zinc Biofortification

The analyses, carried out on the pulp of grapes of the Fernão Pires variety obtained at harvest, detected three soluble sugars: sucrose, glucose, and fructose (Table 3). Grapes, after being sprayed with Zn treatments, did not reveal a significant change in the sugar contents, relative to the control (except for ZnSO_4_ 150 g ha^−1^—Table 3). However, a trend towards higher values of the three sugars in the treatments of ZnSO_4_ and in the maximum dose of ZnO (900 g ha^−1^) was observed (Table 3). Moreover, in general, the most abundant sugars in grapes were glucose and fructose (Table 3), with the values varying between 1.90–2.87, 88.95–182.51, and 94.20–158.84 mg g^−1^ for sucrose, glucose, and fructose, respectively.

### 2.4. Fatty Acid Composition after Zinc Agronomic Biofortification

**Figure 2:**
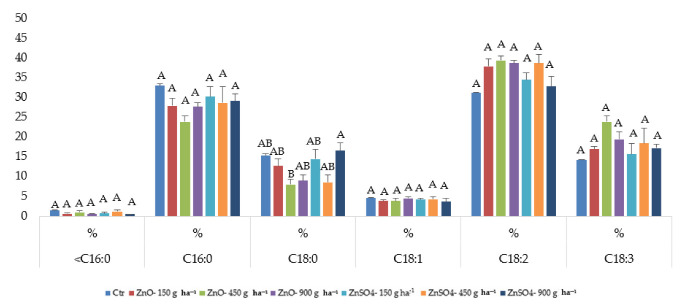
Mean values ± SE (n = 3) of fatty acid profile (mol %) in grape berry lipids of *Vitis vinifera* L. variety Fernão Pires at harvest. Letters A and B indicate significant differences between treatments (statistical analysis using the single-factor ANOVA test, *p* ≤ 0.05).

At harvest, the total fatty acid content (TFA) of the Fernão Pires grapes was not significantly affected through Zn biofortification with ZnO or ZnSO_4_ (Table 4). The degree of unsaturation (DBI) showed a similar pattern, with no detectable differences relative to the control (Table 4). Regarding the fatty acid (FA) profile, the following relative abundance for the control samples was observed: palmitic acid (C16:0) > linoleic acid (C18:2) > stearic acid (C18:0) > linolenic acid (C18:3) > oleic acid (C18:1) > chains inferior to 16 C (<16:0) (Figure 2). In general, with biofortification, FA profiles persisted, despite some changes in percentages, mainly in C16:0, C18:0, C18:2, and C18:3 (Figure 2). The same FA was observed for grapes with ZnO 150, 900 g ha ^−1^, and all treatments of ZnSO_4_ (Figure 2).

### 2.5. Colorimetric Parameters after Zinc Agronomic Biofortification

At harvest, colorimetric analysis of Fernão Pires grapes in the visible spectral region (450–650 nm) did not reveal significant differences among treatments, with maximum values of transmittance found at 550 and 650 nm (Table 5). Therefore, Zn biofortification did not cause relevant variations.

### 2.6. Physical Characteristics and Sensory Analysis

Physical characteristics are important to determine possible differences following Zn spraying that could affect consumers’ acceptability. The results obtained in grapes for firmness, penetration force, and pulp hardness (at harvest) did not show a significant variation, pointing to the absence of negative interference through ZnO and ZnSO_4_ treatments (Table 6). The analyzed parameters varied between 2.93–3.47 N, 8.94–15.57 N*s, and 0.13–0.17 N, for firmness, penetration force, and pulp hardness, respectively (Table 6).

**Figure 3:**
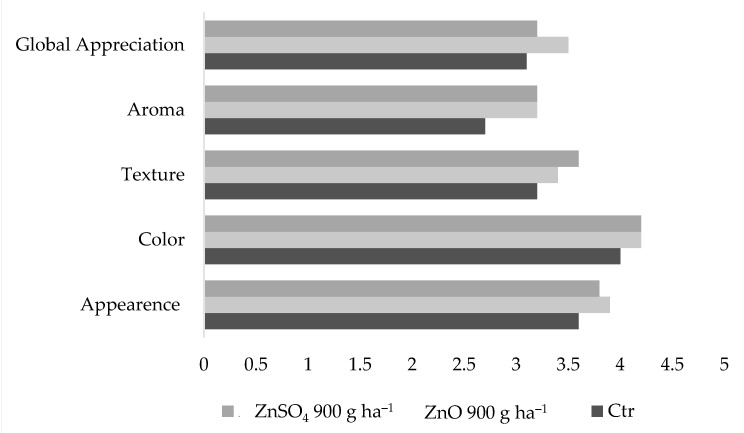
Mean values of sensory parameters of grapes (*Vitis vinifera* L. variety Fernão Pires at harvest in the treatments of 900 g ha^—1^ of ZnO and ZnSO_4_).

Additionally, a sensory test showed a positive response to grapes subjected to Zn foliar application, in global appreciation, aroma, texture, color, and appearance (Figure 3). In fact, a preference for the ZnO-treated grapes (revealing the highest global appreciation) was found (Figure 3).

## 3. Materials and Methods

### 3.1. Experimental Fields

The experimental work was performed in a vineyard of *Vitis vinifera* L. variety Fernão Pires, located in Setúbal, Portugal (GPS coordinates N 38° 35′41.467″ O 8° 50′44.535″ W). Three leaf-spraying applications were performed (after flowering—16 June 7 and 21 July 2018) with ZnO or ZnSO_4_ (150, 450, and 900 g ha^−1^), with the control sprayed with water, only. Harvest was carried out by 17 September 2018. Between 16 June and 17 September, minimum and maximum mean temperatures ranged between 16.6–28 °C.

### 3.2. Field Characterization for Zinc Agronomic Biofortification

**Figure 4:**
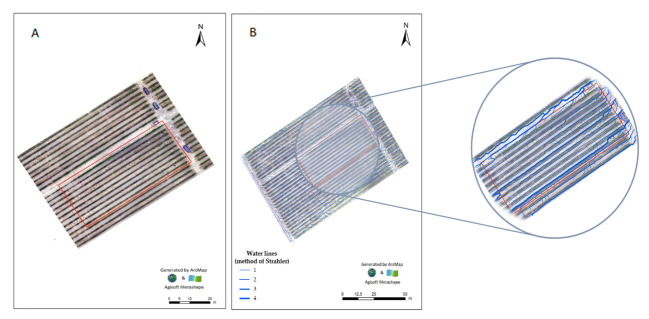
Orthophotomap (**A**) and map of the surface drainage network order of water lines, according to Strahler (**B**), of the vineyard with variety Fernão Pires. Red shows the limits of the field. Information collected before flowering and biofortification treatments.

The vineyard of Fernão Pires has (Figure 4A) an almost flat morphology with a small slope (i.e., variation of 1.10 m between the minimum and the maximum), with a moderate aptitude for accumulation and/or infiltration of surface water (i.e., 61.74%), mostly in the direction of SW–NE (Figure 4B; Table 1).

### 3.3. Orthophotomap and Surface-Water Drainage

Data acquisition occurred on 27 July 2018, using a drone (DJI Phantom 4 Pro +) equipped with high-definition and multi-sector RGB (i.e., with three Electromagnetic Spectra Bands–Red, Green, and Blue) and Parrot Sequoia (i.e., with five Electromagnetic Spectra Bands–NIR, REG, Green, Red, and RGB) cameras. Calibration of the multispectral Parrot Sequoia camera further considered the environmental brightness conditions. Images collected were then processed in a workstation (AORUS, GIGA-BYTE Technology Co., Ltd.-2019), to produce the final orthophotomap. To evaluate the general morphology and surface water drainage areas of the experimental fields, Agisoft PhotoScan Professional (Version 1.2.6, Software of 2016 and the ESRI of 2011 and ArcGIS Desktop-Release 10 from Redlands, CA: Environmental Systems Research Institute) was used. The evaluation of drainage areas of surface waters was carried out as follows [22]. According to the field morphology, the highest class corresponded to the land that enhances the surface runoff of the water and does not promote infiltration. Conversely, the lower class corresponded to flattened surfaces, as potential infiltration areas, since they promote the accumulation of surface water.

### 3.4. Soil Analysis

Soil parameters of the vineyard were determined in 28 samples (approximately 100 g) collected from the surface to a 30 cm depth. Samples were sieved (2.0 mm mesh to remove stones, coarse materials, and other debris), and the weight was recorded after drying (at 105 °C for 24 h, preceded by a 1-h desiccation) for quantification of the dry mass and percentage of moisture. Determination of the content of organic matter was carried out through the heating of samples to 550 °C, for 4 h (i.e., until a constant weight), and was, therefore, removed from the muffle (at 100 °C) and desiccated until at room temperature (approximately 1 h). Samples were then weighed, and the percentage of organic matter was determined. Using a potentiometer, pH and electrical conductivity of soil samples were determined. After mixing, at a ratio of 1:2.5 (g soil mL _water milli-q_), and stirring for 1 h (at 25 °C for 30 min) in a thermal bath, determinations were carried out after decantation of the supernatant [23].

The determination of plant micro and macronutrients in soil samples (n = 28) was performed by using an X-ray analyzer (Thermo Scientific, Niton model XL3t 950 He GOLDD+, Waltham, MA, USA). Detection limits using the optimum mining mode for a period of 120 s under high purity helium (He) were: Ca = 65 µg g^−1^, Fe = 25 µg g^−1^, K = 200 µg g^−1^, Mn = 30 µg g^−1^, S = 90 µg g^−1^, and Zn = 6 µg g^−1^, Soil reference materials (NRCan Till-1) were run before the beginning of analyses and after every five samples [24].

### 3.5. Chlorophyll a Fluorescence

Chlorophyll fluorescence parameters were determined in 4–6 randomized leaves per treatment of *Vitis vinifera* L. variety Fernão Pires (after the 3rd foliar application), using a fluorimeter PAM 2000 (H. Walz, Effeltrich, Germany), as described in [25,26], with some minor modifications. Briefly, minimal fluorescence from the antennae (F_o_) and maximal photochemical efficiency of photosystem (PS) II (F_v_/F_m_) were determined in overnight dark-adapted leaves, applying a low irradiance red light (<0.5 μmol m^−2^ s^−1^), to obtain F_o_, and an actinic saturating light flash of ca. 7500 μmol m^−2^ s^−1^, to obtain maximum fluorescence from the antennae (F_m_), with maximal photochemical efficiency of PSII (F_v_/F_m_), being calculated as ([(F_m_ − F_o_)/F_m_]). Another set of parameters was assessed under photosynthetic steady-state conditions, under natural irradiance (ca. 1000–1300 μmol m^−2^ s^−1^), with superimposed saturating light flashes, which included photochemical quenching, based on the concept of interconnected PSII antennae (q_L_), non-photochemical quenching (q_N_), and the actual PSII photochemical efficiency (F_v_′/F_m_′), as well as the estimates of the quantum yield of photosynthetic non-cyclic electron transport (Y_(II)_), of the quantum yield of regulated energy dissipation of PSII (Y_(NPQ)_), and of non-regulated energy (heat and fluorescence) dissipation of PSII (Y_(NO)_) (with Y_(II)_ + Y_(NPQ)_ + Y_(NO)_ = 1). All parameters and their meanings were obtained based on formulas reported by [27,28,29,30,31].

### 3.6. Analysis of Zn Content in Leaves, Grapes, and Wine

Zn contents in the leaves of *Vitis vinifera* L. variety Fernão Pires were measured, after the 2nd foliar application in randomized leaves (dried at 60 °C, until constant weight, grounded and processed into pellet), following [32]. Zn amount was determined using an XRF analyzer (Thermo Scientific, Niton model XL3t 950 He GOLDD+, Waltham, MA, USA) under He atmosphere. As previously mentioned, the detection limit for Zn is 6 µg g^−1^, while the certified value of orchard leaves (NBS 1571) is 30 µg g^−1^, and the recovery value was 97%.

At harvest, the accumulation of Zn in the tissues of grapes (seeds and skin), was determined with a μ-EDXRF system (M4 Tornado, Bruker, Germany), as described by [33,34], with minor modifications. The X-ray generator was operated at 50 kV and 100 μA without the use of filters, to enhance the ionization of low-Z elements. For a better quantification of Zn, a set of filters between the X-ray tube and the sample, composed of three foils of Al/Ti/Cu (with a thickness of 100/50/25 μm, respectively), was used. All the measurements with filters were performed with a 600 μA current. Detection of fluorescence radiation was carried out by an energy-dispersive silicon drift detector, XFlash, with a 30 mm^2^ sensitive area and energy resolution of 142 eV for Mn Kα. Measurements were carried out under 20 mbar vacuum conditions. These point spectra were obtained for a duration of 200 seconds. Detection limit for Zn is 3 mg kg^−1^. Plant reference materials were used for data validation: orchard leaves (NBS 1571) and poplar leaves (GBW 07604), while the recovery value was 96%.

Zinc content was measured in wine with an atomic absorption spectrophotometer model, the Perkin Elmer AAnalyst 200 (Waltham, MA, USA), fitted with a deuterium background corrector, and using the AA WinLab software program [35,36].

### 3.7. Sugar Analysis

Sugar extraction was performed in 40 g of pulp (removed from several grape berries at harvest) per sample (n = 3), according to [37], with minor modifications. Pulp weighted was liquified with 150 mL of cold ultrapure water and then adjusted to 200 mL. Samples were kept in ice, then transferred to ultrasounds (for 5 min), and later centrifuged (15,000× *g*, 15 min, 4 °C). Thereafter, the supernatant was removed to glass tubes and kept in ice. The pellet was then resuspended in ultrapure water and centrifuged in the same conditions. The supernatant, after the 2nd centrifuge, was transferred to glass tubes (ca. 20 mL) and immersed in a boiling bath (for 4 min). The tubes were then removed and put into ice (for 6 min), and, once cold, the samples were centrifuged (15,000× *g*, 20 min, 4 °C). Samples were injected in an HPLC (Waters, Milford, MA, USA) system, coupled to a refractometric detector (Waters 2414), equipped with a SugarPak 1 column (Waters 6.5 × 300 mm) and pre-column (Wat 088141) with SugarPak II inserts (Wat 015209), after being filtered (nylon 0.45 mm) and stored. Ultrapure water containing 50 ppm calcium EDTA was used as the mobile phase, with a flow of 0.5 mL min^−1^, and an injection volume of 40 µL for each sample. Data were later analyzed with Breeze software, and quantification was performed based on the calibration curves of sucrose, glucose, and fructose.

### 3.8. Fatty Acid Analysis

After harvest, quantitative and qualitative analyzes of fatty acids were carried out in 3–4 bunches composed of several berries without peduncle (ca. 5 g fresh weight) per treatment (3 replicates). After preparation, samples were stored at −20 °C. The fatty acid composition was determined according to [38], by direct acidic transesterification using a methanol:sulfuric acid solution (39:1, *v:v*), after addition of an internal standard (heptadecanoic acid). Esterified fatty acids were analyzed on a gas–liquid chromatograph (Varian CP-3380, Palo Alto, CA, USA), coupled to a flame ionization detector (GC-FID), and separated using a Varian capillary column (CP-Wax 52 CB). The lipid unsaturation index (i.e., the ’double bond index’ (DBI)), reflects the relative abundance of mono and polyunsaturated FAs relatively to saturated FAs, and was calculated using the formula: DBI = ((% monoenes + 2 × % dienes + 3 × % trienes)/% AG saturated), according to [39]. Data were treated using a bivariate ANOVA analysis (*p* ≤ 0.05).

### 3.9. Colorimetric Parameters of Grapes

Colorimetric parameters were determined at harvest, in the pulp of three randomized fresh grapes per treatment (n = 3), with a scanning spectrophotometric colorimeter (Agrosta, European Union). The sensor provides a 40 nm full-width half-max detection, covering the visible region of the electromagnetic spectrum. This sensor has 6 phototransistors with sensibility in a specific region of the spectrum (380 nm—violet; 450 nm—blue; 500 nm—green; 570 nm—yellow; 600 nm—orange; 670 nm—red). Light was furnished by a white light-emitting diode (LED) covering all the visible region [40].

### 3.10. Physical and Sensory Parameters of Grapes

Physical characterization was performed at harvest in the control and treated samples with 900 g ha^−1^ of ZnSO_4_ and ZnO (in order to assess possible changes with higher amounts of Zn), using a texturometer “Texture Analyzer Model TAHDiR” (Stable Micro Systems, Go-dalming, UK), with a 5 kg load cell “Interchangeable Low Force Load Cells Model LC/25”. The test was carried out through penetration in the pulp of the grapes (10 per sample), with a 10 mm diameter aluminum cylindrical probe, at room temperature. Programmed test conditions were: speed 1 mm s^−1^ and probe penetration depth 10 mm. Thus, the force as a function of time graphs was acquired, where the parameters Firmness (N), Penetration work (N * s), and Pulp hardness (N) were calculated [41].

Sensory analyses were performed in the control and 900 g ha^−1^ of ZnSO_4_- and ZnO-treated grapes, considering four randomized grapes and a semi-trained panel of provers (n = 28) in individual cabins, according to NP 4258:1993 (ISO 8589:1988).

### 3.11. Winemaking

Winemaking was carried out with the grapes of the highest concentrations (450 and 900 g ha^−1^ of ZnO or ZnSO_4_), in order to verify possible changes with higher amounts of Zn. Destemming and pressing of the grapes (50 kg) were carried out, thereafter sulfur dioxide was added to the must (18 mL). After 24 h resting at 6 °C, spring aroma (18 g) was added. After 20 min, the yeast was added to the wort after hydration with water at 37 °C (1:10), followed by homogenization of the mixture. The temperature and density of the mixture were then regularly checked. PVPP/Polyvinylpolypyrrolidone–Divergan F (12 g) was applied when the density reached 1060 g/cm^3^, and DAP–Diammonium phosphate (12 g) was applied at the peak of fermentation (density between 1030–1040 g/cm^3^) and when the density reached 1000 g/cm^3^. Sulfur dioxide (3 mL) was further added when the density reached 990 g/cm^3^. The wine was then filter and bottled.

### 3.12. Statistical Analysis

Data were statistically analyzed using a one-way or two-way ANOVA (*p* ≤ 0.05), to evaluate differences between treatments and experimental periods, followed by a Tukey’s test for mean comparison, considering a 95% confidence level. For the two-way ANOVA, letters (A, B, C or a, b, c) indicate significant differences between treatments for the same date, or between dates for each treatment, respectively.

## 4. Discussion

The effects of climatic conditions and soil type on grape ripening and wine quality in two Cabernet Sauvignon vineyards under the same climate, but on distinct soils, revealed that soil type is determinant in wine phenolic composition and tasting characteristics [42]. In fact, the soil influences vine development and grape ripening through soil temperature, water supply, and mineral supply. Soil temperature has a significant effect on vine phenology, while a shortage in the water supply restricts shoot and berry growth, which is critical for reaching a suitable grape composition to produce high-quality red wines [43]. In our case, the vineyard with variety Fernão Pires expands through a slight slope with low or moderate surface drainage (i.e., classes 1 and 2), with a homogeneous water distribution, but with a higher propensity for accumulation and/or infiltration of surface water in the SW–NE direction. Unfortunately, the current severe drought in mainland Portugal might well affect grape sugar, as noted by Van Leeuwen et al. [44], although phenolic compounds and particularly anthocyanins could be increased in grape skins [45].

Soil fertility and soil physical and chemical characteristics are of great importance regarding to grape quality. For example, the presence of soil carbonates in European vineyards probably leads to deficiencies in some nutrients such as Mn, reducing the availability to vines, and affecting the color in red grapes [46]. In the same context, Bramley et al. [47] claimed that the availability of Fe and Mn in adequate quantities is very important for producing high-quality wines, although emphasizing the importance of N fertilization, which is responsible for both a vine´s vigor and yield, when applied at moderate levels, since the excess of N slows down the process of maturation, producing juices with fewer sugars and phenolic compounds.

Our current levels of Mn in soil vineyards (299 mg kg^−1^) are compared with other data from the Iberian Peninsula, in which higher levels were observed in the Castilla-La Mancha vineyards, despite the huge variation found in the soils, i.e., 380 mg kg^−1^ ± 740 [48]. The mean concentration in vineyards soils from northwest Romania was closer to ours i.e., 250 mg kg^−1^ [48]. Regarding Fe, the concentrations of both origins were almost identical—22 g kg^−1^ and 21 g kg^−1^ from Spain and Romania, respectively [48,49], while from the present study only 3.5 g kg^−1^ was detected. Similar concentrations were also found between Ca levels in Romanian soils [49] and the average value observed by us, i.e., 5.3 g kg^−1^ and 5.4 g kg^−1^, respectively.

Evaluation of total macro- and micronutrients in soils is not a good predictor of the concentration in the plants [50], since the plant-available fraction is a function of soil characteristics, mainly the pH and organic matter. In this context, several authors use the available levels of N, P, and K, mainly, among other elements, for establishing relationships with the elemental composition of the grapes and wines [51,52]. For example, Mackenzie and Christy [53], when studying the elemental composition of soil vineyards, observed that grape juice properties such as Baumé/Brix and titratable acidity are clearly correlated with several plant-available trace elements in the soil, mainly Ca, Sr, Ba, Pb, and Si. Nevertheless, some criticism exists about the multiple extraction-procedure methods to determine the available fraction [54]. Despite this, different authors consider that the mineral composition pattern is transferred through the soil–wine system, and differences observed for soils are reflected in grape musts and wines, though not for all elements [48,49,55]. In this framework, it must be emphasized that evaluation of soil macro- or micronutrients as soil quality indicators is not recommended in Australia, because current industry practice utilizes petiole analysis for macro- and micronutrient testing rather than soil tests, and negligible soil data are available [56]. It is well-known that petiole sampling is useful for the posterior analysis and diagnosis of the nutritional state of vineyards at field level [46].

As previously stated, soil chemical characteristics are of great importance in crop production and, particularly, the effect of soil pH on the availability of nutrients to grapevines. The optimum pH range (measured in water) for nutrient uptake is between 5.5 and 8 [57], thus, our pH value of 6.85 falls within the ideal range mentioned above. Despite the lack of an adequate threshold of soil organic matter for viticulture, a level of 2% is recommended for Australian soils [58], although its importance in maintaining soil structure varied vis a vis the type of soil vineyard. Levels of soil organic matter ranging between 1–1.59% are considered as a moderate rate [58]. The levels observed by us in a previous work [18] in the same region with two different grape varieties (Moscatel and Castelão) gave us 1.09–1.48%, which is in agreement with the current value observed—1.36%.

The Zn content of grape seeds collected from 50 different locations of Turkey was studied by inductively coupled plasma atomic emission spectrometry. The results show that the average levels varied between a minimum of 6.5 and a maximum of 25.6 mg kg^−1^, although the majority of the concentrations range between 10 and 14 mg kg^−1^, which encompasses 56% of the samples [59]. As expected, our biofortified grape seeds exhibit higher Zn values, and our control grape seeds have a level close to the maximum referred above, i.e., 24.6 mg kg^−1^.

In the current study, the Zn content of non-biofortified wine (control) is 0.98 mg L^−1^, while the maximum average value was 1.56 mg L^−1^ observed in the treatment with ZnSO_4_, at a foliar spraying rate of 450 g ha^−1^. Two different vineyards from the Douro wine district, Portugal, were studied regarding the multielement composition of wines. The first one was from a 10-year-old vineyard (monovarietal grapes) and was used to produce a red table wine, while the second one was from a 60–70-year-old vineyard (polyvarietal) and was used to produce a red fortified wine, similar to the world-famous Port wine [52]. The authors concluded that the fortified wine has a Zn level of 1.0 mg L^−1^, while the red table wine has only 0.43 mg L^−1^. In Croatia, the contents of several selected metals in both red and white wine samples (n = 70) were collected from the continental (northeast) and Adriatic areas (near the Adriatic Sea) and were determined by total reflection X-ray analysis fluorescence spectrometry (TXRF). The levels of Zn range between 0.51–1.01 mg L^−1^, although it must be stressed that grapes were not submitted to foliar Zn fertilization [60]. All these values fell in the ranges frequently found in different wines for Zn, i.e., between 0.5 and 3.5 mg L^−1^ [61].

It was reported that Zn is involved in the biosynthesis of chlorophyll, and, under plant stress, the inhibition of the electron transport chain prevails [10]. In the current study, after three foliar applications, no significant changes on chlorophyll a parameters occurred (Fv/Fm, Y(_NO_), Y(_II_), Y (_NPQ_), qL, qN, and Fv′/Fm′) between treatments, indicating that the threshold of toxicity was not reached, despite our measuring almost 500 mg.kg^−1^ in the leaves with the highest concentration of ZnSO_4_ (900 g ha^−1^), much above the limit of 300 mg.kg^−1^, as indicated by [1], which means that the latter value is an average indication and does not encompass, obviously, the hyperaccumulator Zn plants. However, during fruit development, a higher energy is necessary for biomass production compared to the final stage of the production cycle [62], as it was observed in general for Y(_II_) and qL, when comparing the two monitored dates (Figure 1). Moreover, no significant variations for qN and Y(_NPQ_) were noted, despite that the values tended to increase at the second assessment, which may be related to the production cycle. Indeed, in earlier stages, the photoprotective mechanism of the photosynthetic machinery is more active, as capacity was progressively lost while the berries grow [61].

Considering the harvest time, vines are more susceptible to additional stresses [63,64], namely due to high temperature or drought that can lead to increased synthesis of reactive oxygen species (ROS) and, consequently, to a reduction in photosynthetic CO_2_ fixation [65,66]. Y(_NPQ_) and qN are related to a photoprotective mechanism that prevent photo-oxidative stress in plants, which means that the increase in these values in the second assessment indicates a higher need for energy dissipation, via the xanthophyll cycle, to minimize potential damage to the thylakoid membranes [67]. After the third application, the content of Zn was higher in treated leaves, as expected, although at harvest part of the Zn load had been mobilized to the grape itself, where seed and skin are included (Table 2). In fact, Zn is involved in grape development due to the synthesis of growth regulators and chlorophyll, although it is dependent on the ripening stage [68].

According to Christensen [69], Zn is very mobile and its solubility did not influence absorption after foliar spraying in vineyards—low-solubility neutral zinc and ZnO gave responses similar to the other fully soluble compounds, and, at the rates that are normally used, none of the compounds caused visible vine-foliage toxicity. In our case, the highest Zn leaf levels were observed with ZnSO_4_, although, when the highest concentration was applied, the concentration in the seed and grape skin decreases. Conversely, the highest level of ZnO did not decrease in both the seeds and skin, despite the accumulation in the leaves being clearly lower. These apparent interesting results can be negatively influenced by the winemaking process (i.e., maceration, extraction, and solubilization during fermentation), where mineral losses occurred, which was confirmed in the present study. Depletion of some elements occurs over time, especially during alcoholic fermentation—for example, the precipitation of K and Ca as tartrate salts begins during alcoholic fermentation and continues during the aging period [55].

Sucrose, glucose, and fructose are main components in grapes, with an important role in consumers’ acceptability, mostly due to the interaction among the sugar content, synthesis of organic acids and phenolics, sensory properties, alcohol concentration in winemaking, and aroma compounds [70,71,72]. At harvest, the Zn-biofortified grapes of Fernão Pires with ZnSO_4_ and ZnO do not present relevant variations of sucrose, glucose, and fructose contents. As sucrose is the final product of photosynthesis, our data further indicate the absence of inhibitory effects in Zn-treated vines. Additionally, the hydrolysis of sucrose, after being produced in leaf photosynthesis and transported through the phloem to the berry, determined the similar levels of the isomers of glucose and fructose. Besides, our data (Table 3) agree with [70,72], where glucose and fructose present higher values compared to sucrose, and the amount of fructose was slightly superior to glucose. In general, grapes treated with ZnSO_4_ and ZnO showed a tendency for a higher sugar content (except for ZnO 150 and ZnO 450 g ha^−1^ for sucrose and glucose/fructose, respectively), which is an important quality parameter for winemaking, as it affects the fermentation process and alcohol contents [73], although we must note that a high alcohol content can causes a gustatory disequilibrium affecting wine sensory perceptions, leading to unbalanced wines [74].

The data from the total fatty acid (TFA’ levels from the current study clearly show that ZnSO_4_ has a more pronounced effect on grapes than ZnO, and, in some cases, the levels are even lower than the control grapes (Table 4), which may be important regarding the profile of the wine. The manipulation of TFA levels is a goal of some researchers through the use of exogenous compounds, such as abscisic acid and methyl jasmonate, in order to promote the enrichment of polyunsaturated fatty acids and/or monounsaturated fatty acids [75], which was not our goal. Polyunsaturated fatty acids and phenolic compounds are important molecules in grapes, due to their role in the prevention of cardiovascular disease and antioxidant potential, respectively [76]. Fatty acid composition in grapes of Fernão Pires (Figure 2; Table 4) revealed a higher presence of acid linoleic (C18:2), acid palmitic (C16:0), stearic acids (C18:0), and linolenic acid (C18:3), which have a potential influence in the aroma production during winemaking, as observed for the cultivar Cabernet Sauvignon grape [77].

The colorimetric analyses also did not show visible changes (Table 5), which indicates that Zn treatments (i.e., 150–900 g ha^−1^ of ZnSO_4_ and ZnO) did not influence the relative proportions of chlorophylls and carotenoids that determine the final white grape berry color [78], as well as the color of the wine, which is an important aspect for consumers [79]. Besides, the physical characteristics of Zn-treated grapes did not reveal an evident negative impact (Table 6), which is of the utmost importance, since grapes’ size, appearance, texture, and sensory characteristics are important for commercialization [80]. In fact, sensorially Zn-treated grapes were the most appreciable, pointing to an absence of an undesirable organoleptic response to ZnSO_4_ and ZnO (Figure 3).

## 5. Conclusions

Zinc biofortification with ZnO and ZnSO_4_ at concentrations of 150, 450, and 900 g ha^−1^ effectively increased the Zn amount in tissues of *Vitis vinifera* variety Fernão Pires, but the photosynthetic performance was not significantly affected, which showed a potential benefit for grapes production. After winemaking, losses in Zn occurred, related to the different wine production stages, but Zn values remained significantly higher relative to the control wine. Besides, Zn accumulation did not substantially affect the physicochemical characteristics of grapes (sugars, fatty acids, color, and texture), which indicates that Zn-sprayed vines benefits growth and development, without a negative impact in the quality parameters.

## Figures and Tables

**Table 1:** Characterization of soil (at 0–30 cm deep) in the vineyard of Lau Velho field.

Aptitude to Accumulate or Drain Surface Water									
Slope Class (%)			Surface Drainage		Area (m^2^)		Área (%)		
1—[0–5%]			Low		682.7		34.43		
2—[5–20%]			Moderate		1224.3.4		61.74		
3—>20%			High		76.1		3.84		
**Soil Analysis (0–30 cm deep) (n = 28)**									
pH	Electrical Conductivity	Organic Matter	Ca	K	P	Fe	S	Zn	Mn
	μS cm ^−1^	%					ppm		
6.85 ± 0.07	93.4 ± 17.78	1.36 ± 0.15	0.54 ± 0.15	3.53 ± 0.09	0.17 ± 0.02	0.35 ± 0.02	305.6 ± 117.8	58.9 ± 8.99	299.0 ± 43.3

**Table 2:** Mean values ± SE (n = 3) of Zn concentration of *Vitis vinifera* L. variety Fernão Pires in the leaves after the 3rd foliar application, as well as in the seed and skin of grapes at harvest and in the produced wine. Letters A, B, C, D, and E indicate significant differences within the same column and variety (statistical analysis using the single-factor ANOVA test, *p* ≤ 0.05).

	Zn Content			
	Leaves (ppm)	Grapes (ppm)		Wine (mg L^−1^)
Treatments		Seed	Skin	
**Ctr**	32.76 ± 4.36 E	24.6 ± 1.23 D	23.4 ± 1.17 D	0.98 ± 0.01 B
**ZnO (150 g ha^−1^)**	110.30 ± 1.39 C,D	17.1 ± 0.85 E	37.5 ± 1.88 C	-
**ZnO (450 g ha^−1^)**	176.98 ± 32.37 C	37.1 ± 1.85 B,C	54.4 ± 2.72 A,B	1.45 ± 0.05 A
**ZnO (900 g ha^−1^)**	337.72 ± 0.00 B	44.7 ± 2.24 A	59.4 ± 2.97 A	1.53 ± 0.03 A
**ZnSO_4_ (150 g ha^−1^)**	91.75 ± 13.45 D,E	49.9 ± 2.49 A	54.2 ± 2.71 A,B	-
**ZnSO_4_ (450 g ha^−1^)**	297.47 ± 20.03 B	42.9 ± 2.15 A,B	50.0 ± 2.50 B	1.56 ± 0.02 A
**ZnSO_4_ (900 g ha^−1^)**	496.22 ± 4.57 A	30.1 ± 1.5 C,D	39.8 ± 1.99 C	1.44 ± 0.11 A

**Table 3:** Mean values ± SE (n = 3) of sugar content in grapes of *Vitis vinifera* variety Fernão Pires at harvest. Letters A and B indicate significant differences (statistical analysis using the single-factor ANOVA test, *p* ≤ 0.05).

Sugar Content in Grapes (mg g^−1^ Fresh Weight)			
Treatments	Sucrose	Glucose	Fructose
**Ctr**	2.13 ± 0.61 A	102.12 ± 21.34 B	102.78 ± 21.88 A
**ZnO (150 g ha^−1^)**	1.90 ± 0.20 A	111.07 ± 25.24 A,B	116.96 ± 26.04 A
**ZnO (450 g ha^−1^)**	2.85 ± 0.28 A	88.95 ± 5.490 B	94.20 ± 6.45 A
**ZnO (900 g ha^−1^)**	2.77 ± 0.45 A	132.92 ± 20.82 A,B	135.20 ± 19.77 A
**ZnSO_4_ (150 g ha^−1^)**	3.35 ± 0.51 A	182.51 ± 11.25 A	158.84 ± 20.23 A
**ZnSO_4_ (450 g ha^−1^)**	2.87 ± 0.58 A	130.18 ± 11.07 A,B	135.43 ± 14.24 A
**ZnSO_4_ (900 g ha^−1^)**	2.72 ± 1.19 A	134.85 ± 6.91 A,B	140.19 ± 5.79 A

**Table 4:** Mean values ± SE (n = 3) of total fatty acid content (TFA g/100 g) and insaturation (DBI, double bond index) of grape berry lipids of *Vitis vinifera* L. variety Fernão Pires at harvest. Letter A indicates the absence of significant differences between treatments (statistical analysis using the single-factor ANOVA test, *p* ≤ 0.05).

Treatments	TFA	DBI
**Ctr**	0.91 ± 0.05 A	2.21 ± 0.01 A
**ZnO (150 g ha^−1^)**	1.05 ± 0.11 A	3.21 ± 0.34 A
**ZnO (450 g ha^−1^)**	1.35 ± 0.21 A	4.73 ± 0.21 A
**ZnO (900 g ha^−1^)**	1.12 ± 0.18 A	3.83 ± 0.43 A
**ZnSO_4_ (150 g ha^−1^)**	0.81 ± 0.04 A	2.76 ± 0.57 A
**ZnSO_4_ (450 g ha^−1^)**	1.06 ± 0.07 A	4.00 ± 1.30 A
**ZnSO_4_ (900 g ha^−1^)**	0.54 ± 0.04 A	2.69 ± 0.42 A

**Table 5:** Mean values ± SE (n = 3) of the transmittance of visible spectra in grapes of *Vitis vinifera* L. variety Fernão Pires at harvest. Letter A indicates absence of significant differences (statistical analysis using the single-factor ANOVA test, *p* ≤ 0.05).

Transmittance (nm)						
Treatments	450	500	550	570	600	650
**Ctr**	616.00 ± 9.54 A	522.67 ± 5.21 A	886.00 ± 10.41 A	465.33 ± 3.93 A	546.00 ± 4.16 A	902.33 ± 6.69 A
**ZnO (150 g ha^−1^)**	491.00 ± 106.17 A	390.33 ± 100.83 A	679.00 ± 141.57 A	362.33 ± 71.67 A	458.33 ± 68.41 A	783.00 ± 96.17 A
**ZnO (450 g ha^−1^)**	555.67 ± 12.99 A	467.00 ± 9.64 A	808.00 ± 19.30 A	415.33 ± 2.73 A	498.33 ± 8.29 A	842.67 ± 8.57 A
**ZnO (900 g ha^−1^)**	451.00 ± 71.14 A	362.33 ± 61.20 A	671.67 ± 42.78 A	344.00 ± 28.84 A	382.00 ± 29.50 A	702.33 ± 69.63 A
**ZnSO_4_ (150 g ha^−1^)**	568.33 ± 44.28 A	466.00 ± 47.43 A	767.00 ± 115.11 A	405.00 ± 52.21 A	470.00 ± 62.39 A	839.67 ± 42.45 A
**ZnSO_4_ (450 g ha^−1^)**	576.00 ± 42.36 A	482.00 ± 36.30 A	867.67 ± 37.26 A	458.67 ± 24.01 A	517.00 ± 30.07 A	873.00 ± 42.51 A
**ZnSO_4_ (900 g ha^−1^)**	530.00 ± 62.60 A	422.67 ± 58.81 A	720.67 ± 36.20 A	377.33 ± 28.70 A	436.67 ± 29.08 A	779.33 ± 54.81 A

**Table 6:** Mean values ± SE (n = 3) of firmness, penetration force, and pulp hardness in *Vitis vinifera* L. grapes variety Fernão Pires. Letter A indicates the absence of significant differences between treatments (statistical analysis using the single-factor ANOVA test, *p* ≤ 0.05).

Physical Characteristics			
Treatments	Firmness [N]	Penetration Force [N·s]	Pulp Hardness [N]
Ctr	3.47 ± 0.32 A	15.57 ± 4.47 A	0.13 ± 0.03 A
ZnO (900 g ha^−1^)	3.27 ± 0.62 A	11.46 ± 2.15 A	0.13 ± 0.05 A
ZnSO_4_ (900 g ha^−1^)	2.93 ± 0.47 A	8.94 ± 2.19 A	0.17 ± 0.05 A

## Data Availability

Not applicable.
